# Non-linear dose-response association between physical activity and mental health in adolescents: a prospective cohort study based on SEARCH

**DOI:** 10.1186/s12966-025-01848-y

**Published:** 2025-11-20

**Authors:** Yue Zhang, Zhuo Wang, Ran Zhang, Yang Wang, Jie Yang, Fei Wang, Zhuang Liu

**Affiliations:** 1 School of Public Health, China Medical University, No.77, Puhe Road, Shenbei New District, Shenyang, Liaoning 110122 China; 2 College of Exercise and Health, Shenyang Sport University, No.36 Jinqiansong East Road, Sujiatun District, Shenyang, 110102 China; 3 Early Intervention Unit, Department of Psychiatry, The Affiliated Brain Hospital of Nanjing Medical University, No.264 Guangzhou Road, Nanjing, Jiangsu 210029 PR China; 4 Functional Brain Imaging Institute of Nanjing Medical University, No.264 Guangzhou Road, Nanjing, Jiangsu 210029 China; 5 Jiangsu Provincial Center for Disease Control and Prevention, No.172, Jiangsu Road, Gulou District, Nanjing, Jiangsu 211000 China; 6 School of Public Health, Nanjing Medical University, No.101, Longmian Avenue, Jiangning District, Nanjing, Jiangsu 211166 China; 7 Department of Mental Health, School of Public Health, Nanjing Medical University, No.101, Longmian Avenue, Jiangning District, Nanjing, 211166 China

**Keywords:** Adolescents, Mental health, Physical activity, Duration

## Abstract

**Background:**

Adolescent mental health has emerged as a critical public health issue, with increasing prevalence of anxiety and depression. Physical activity (PA) is known to offer numerous health benefits, but its relationship with adolescent mental health is less clear and the optimal intensity and duration remain debated.

**Methods:**

This prospective cohort study analyzed data from 6,991 adolescents in Jiangsu, China. Mental health was assessed using the Strengths and Difficulties Questionnaire (SDQ), and PA was categorized as moderate-intensity PA (MPA) or vigorous-intensity PA (VPA) based on self-reported activity levels. The study examined the cross-sectional and longitudinal associations between PA and mental health, adjusting for various confounding factors.

**Results:**

Among the cohort (mean age, 12.71 ± 2.12 years; 53.6% male), 1,265 participants (18.1%) had mental health issues at baseline. Cross-sectional analyses revealed a significant negative non-linear dose-response association between levels of MPA and VPA and the prevalence of mental health problems in adolescents (*P* = 0.001), after adjusting for potential confounding factors such as age, gender, nationality, and family structure. Specifically, adolescents who engaged in 30–59 min of MPA daily exhibited a 56.4% reduction in the odds ratios of mental health issues (OR = 0.436, 95% CI: 0.327–0.581, *P* < 0.001). Similarly, those who participated in up to 29 min of VPA daily had 49.2% lower odds ratios (OR = 0.508, 95% CI: 0.415–0.622, *P* < 0.001). Longitudinal analyses confirmed that MPA and VPA were significantly negatively associated with the incidence of subsequent mental health problems. In particular, adolescents who engaged in 30–59 min of MPA daily (OR = 0.548, 95% CI: 0.411–0.729, *P* < 0.001) and up to 29 min of VPA daily (OR = 0.583, 95% CI: 0.474–0.716, *P* < 0.001) demonstrated the strongest protective effects. Sensitivity analyses and subgroup analyses also confirmed the robustness of these results.

**Conclusions:**

Significant positive associations exist between moderate PA and improved adolescent mental health outcomes. The better effects are observed with 30–59 min of MPA or ≤ 29 min of VPA daily. Exceeding these durations may not yield additional benefits. When promoting PA among adolescents, focus should be placed on selecting appropriate activity types and scientifically managing activity duration.

**Supplementary Information:**

The online version contains supplementary material available at 10.1186/s12966-025-01848-y.

## Background

Mental health disorders have increasingly been recognized as a critical global public health issue [1]. The onset of these disorders has been documented to significantly affect an individual’s cognitive capacities, behavior, interpersonal relationships, physical functioning, and overall life satisfaction. Empirical research indicates that mental health challenges affect 25% to 30% of adolescents and young adults [2, 3]. A previous review of 43 studies on common mental disorders (CMD) in adolescents reported a global prevalence of 25.0% and 31.0%, contingent upon the use of the General Health Questionnaire (GHQ-12) cut-off point of 4 or 3, respectively [4]. These adolescent mental health issues can severely hinder healthy development, leading to substantial psychological and social repercussions in adulthood. On December 31, 2019, the World Health Organization (WHO) declared the outbreak of the novel coronavirus as an international public health emergency. Since the initial emergence of the outbreak, the virus has disseminated across more than 200 countries and regions, resulting in the infection of hundreds of thousands of individuals. The ramifications of these events have been profound, engendering widespread panic and mental health distress [5, 6]. Stressful events are potentially adverse environmental factors that can predispose individuals to mental illnesses, particularly depression [7, 8]. Adolescents, in particular, constitute a vulnerable demographic; the disruptions caused by school closures and the pandemic have exacerbated adolescent mental health issues, compounded by increased screen time, social media usage, economic instability, and the morbidity and mortality associated with COVID-19 [9, 10]. Prior to the advent of the COVID-19 pandemic, the global prevalence of mental health issues among adolescents had reached 20% [1]. Since the outbreak of the COVID-19 pandemic, the prevalence rates of anxiety and depression among Chinese adolescents have shown a significant upward trend. Notably, among high school students, their self-reported rates of anxiety and depression reached 37.4% and 43.7%, respectively [11]. This alarming increase in mental health disorders among adolescents is adversely affecting their daily functioning, academic performance, and interpersonal relationships. Consequently, adolescent mental health has emerged as a critical area of research and public concern.

Participation in physical activity (PA) is associated with numerous health benefits, such as a reduction in all-cause mortality, enhancements in musculoskeletal health and stress regulation, and a decreased risk of developing cardiovascular disease, obesity, stroke, and cancer [12, 13]. WHO advises that adolescents aged 5 to 17 years should engage in at least 60 min of moderate- vigorous-intensity PA (MVPA) on a minimum of three days per week [14]. Additionally, PA has been integrated into treatment guidelines for various diseases, such as type 2 diabetes [15], osteoarthritis [16], osteoporosis [17], sarcopenia [18], among others. Childhood and adolescence are critical periods marked by rapid growth and development, including neuronal plasticity [19] and the formation of behavioral patterns that can either support or hinder mental health [20]. It is clear that PA is crucial in fostering growth and development during these stages. Moreover, a significant correlation exists between adolescent mental health and PA. Numerous studies have shown that adolescents who participate in PA experience reductions in anxiety and depression [21], improvements in academic performance [22], decreases in anger and psychological distress, and increases in life satisfaction [23]. Cross-sectional studies have demonstrated that adherence to aerobic PA and muscle-strengthening exercise (MSE) recommendations is associated with a decreased prevalence of mental health issues [24]. Several randomized controlled trials (RCTs) conducted by various research teams have corroborated the beneficial effects of PA on psychological issues across diverse adolescent populations [25–27]. Additionally, cohort studies have shown that substituting screen time with physical exercise positively influences emotional well-being [28]; and that increased PA directly contributes to the reduction of social anxiety by bolstering adolescents’ psychological resilience [29]. However, the relationship between exercise and adolescent mental health continues to be a subject of debate. The impact of various exercise modalities, intensities, and durations on adolescent mental health remains contentious. Critically, MPA and VPA may influence mental health through distinct physiological pathways and thus exhibit different dose-response relationships. MPA (e.g., brisk walking, cycling), typically elevates heart rate to 50–70% of maximum [30]. This moderate intensity facilitates prolonged duration and primarily enhances aerobic capacity and cardiovascular efficiency. In contrast, VPA requires heart rates exceeding 70% of maximum [30]. Its higher intensity robustly stimulates the release of key neurochemicals like brain-derived neurotrophic factor (BDNF) and endorphins, which are intimately linked to mood regulation and stress resilience [31–33]. This pronounced neurochemical response may underlie the effects of VPA on specific mental health outcomes. Consequently, it is essential to investigate the relationship between different exercise intensities and durations and mental health issues in adolescents, utilizing a substantial sample size. Certain RCTs have substantiated the therapeutic benefits of moderate exercise for individuals experiencing mild to moderate depression [34–36], whereas other studies have failed to corroborate these findings [37, 38]. Additionally, the physiological effects of exercise are intricate, and it is yet to be determined whether increased exercise dosages yield greater benefits. It is therefore essential to investigate the relationship between different exercise intensities and durations and mental health issues in adolescents, employing a large sample size.

Given the potential for distinct effects of MPA and VPA, and to move beyond the limitations of previous studies that often combine them, our analysis deliberately examines these intensities separately. From a public health and research perspective, identifying the risk factors for adolescent mental health problems is crucial for developing effective management strategies and prevention or treatment interventions. The etiology of adolescent mental health issues is widely recognized as multifaceted, involving complex interactions between genetic predisposition and environmental factors [39, 40]. Although numerous studies have explored various potential influencing factors, such as gender [41], age [42, 43], economic stress, family structure, sleep patterns [44], smoking, alcohol consumption [45], and screen time [46], no single factor can fully explain the occurrence and development of mental health problems in this population. Recognizing this complexity, focusing on modifiable behaviors like PA holds significant scientific and practical value. This is primarily because it serves as a modifiable target for public health interventions, offering advantages in terms of being adjustable, scalable, and broadly applicable across populations. While acknowledging the multifactorial nature of adolescent mental health problems, we chose to focus on PA because it represents a key modifiable behavior with the potential to promote mental health both independently and in conjunction with other factors. This research design allows us to delve into a specific and actionable component within a more complex etiological landscape.

Substantial evidence indicates an association between PA and improved adolescent mental health. Cross-sectional studies [24] and longitudinal cohort studies [28, 29] consistently reveal the protective effects of PA, while RCTs [25–27] have confirmed the effectiveness of exercise as a method for enhancing mental health. However, existing research is often limited by sample size, and few studies have systematically examined the effects of different exercise intensities, frequencies, and durations. In particular, the dose-response relationship between MPA and VPA and mental health outcomes remains incompletely understood, this is the critical gap that the present study aims to fill.

To address this, our study utilized a cohort dataset comprising nearly 7,000 adolescents to comprehensively investigate the impact of MPA and VPA on their mental health. The core innovations of this study are twofold: first, establishing the dose-response relationship between PA and adolescent mental health problems; second, identifying the optimal timing for engaging in PA of different intensities to alleviate these problems. We hope that this research will provide a theoretical basis for developing more targeted exercise prescriptions, thereby effectively addressing the mental health challenges faced by adolescents.

## Methods

### Study design and participants

This study utilizes data from the School-based Evaluation of Responses to Child Health Promotion (SEARCH) research conducted in China [47]. This cohort study was implemented within the school environment and was spearheaded by the Institute of Child and Adolescent Health Promotion at the Jiangsu Provincial Center for Disease Control and Prevention. The institute was responsible for site selection and overall coordination, while local field investigators managed the specific data collection tasks. This research initiative facilitated the establishment of the “School-based Alliance for Children and Adolescents’ Mental Health”, which fostered collaboration among representatives from the Jiangsu Provincial Central Research Institute, local field investigators, and members of the project supervision team. The SEARCH study is a longitudinal cohort study designed to monitor the mental health of Chinese adolescents. To ensure that the research can reflect the situations of regions with different levels of economic development, we carefully selected three cities in Jiangsu Province, namely Sheyang, Yixing and Taizhou, which have different levels of economic development, as the research sites. This study recruited adolescents from grades 4–6 of primary school, grades 7–9 of junior high school and grades 10–12 of senior high school in Jiangsu Province as the research subjects. Baseline data were collected from April to May 2023, enrolling a cohort of 12,053 adolescents. The follow-up survey was conducted from October to December 2023. Given that the cohort included students in critical transition years, a high attrition rate was anticipated due to normal academic progression, which precluded continued participation. Consequently, 5,062 participants from the original cohort were lost to follow-up (a 42.0% loss rate). The final analytical sample consisted of 6,991 adolescents who completed both survey waves. In accordance with the detailed study protocol, informed consent and assent were obtained from all participants and their legal guardians. The study received approval from the Ethics Committee of the Affiliated Brain Hospital of Nanjing Medical University (Approval No. 2022-KY095-02). All participants provided informed consent prior to participation, and for minors, consent was obtained from their legal guardians. All procedures adhered to the Strengthening the Reporting of Observational Studies in Epidemiology (STROBE) guidelines (see Online Supplemental Checklist).

### Mental health and PA assessment

Mental health issues were evaluated utilizing the Strengths and Difficulties Questionnaire (SDQ), a validated instrument demonstrating strong concurrent validity among Chinese adolescents [48]. The SDQ is a concise questionnaire consisting of five subscales: hyperactivity, behavioral problems, peer problems, emotional problems, and prosocial behaviors. It has been demonstrated to possess good psychometric properties, making it a useful tool for assessing the adaptation status and psychopathology of children and adolescents [49]. In this research, a total difficulties score of 0–15 was defined as “healthy”, while >15 was classified as “unhealthy” based on validated thresholds recommended by SDQ developers [50, 51] and confirmed in Chinese adolescent populations [52].

PA was assessed using a self-reported measurement instrument, whereby participants provided detailed accounts of their physical activities over a seven-day period, specifying the number of hours engaged in such activities per day. The self-reported physical activities were classified into two categories: MPA and VPA. MPA, including brisk walking, cycling, jogging, dancing, Tai Chi, and yoga, are distinguished by a modest elevation in breathing and heart rate. Engagement in VPA, such as long-distance running, jumping rope, and participating in sports like soccer, basketball, tennis, badminton, table tennis, and swimming, leads to a significant increase in both respiratory and heart rates. The durations of MPA and VPA were classified into four distinct categories based on the WHO guidelines for adolescent PA: 0 indicating no activity, 1 representing up to 29 min per day, 2 for 30 to 59 min per day, and 3 for 60 min or more per day.

Given the inherent limitations of self-report methods, which can lead to biases that either underestimate or overestimate actual activity levels, we implemented the following strategies to mitigate potential bias. First, the questionnaire provided clear and specific definitions and examples for each type of activity (MPA and VPA), aiming to enhance participants’ understanding of the activity types and thereby improve the consistency of their reports. Second, we asked participants to recall their activities over the past 7 days; compared to longer recall periods, this relatively short time window helps to minimize recall bias. Finally, while acknowledging the inherent limitations of self-reporting, we adjusted for relevant covariates in our statistical models, such as age, gender, and socioeconomic status. These covariates may be related to reporting bias and PA levels, and by adjusting for them, we attempted to control for some of the variance attributable to measurement error. Additionally, using the WHO guidelines to categorize activity duration also helps reduce the impact of minor inaccuracies in self-reported time.

### Assessment of covariates

To accurately assess the association between PA and mental health outcomes while minimizing the influence of confounding factors, we incorporated a range of potential confounders into our analysis. The adjustment covariates were selected in advance using a directed acyclic graph (DAG) (online Supplemental Figure S1). These covariates included demographic factors (age, biological gender, nationality, family structure, economic region, frequency of family conflicts), a health-related measure [body mass index (BMI)], and lifestyle factors (smoking, drinking). To minimize potential mediating effects and ensure temporal precedence, the analysis utilized covariate data collected exclusively at baseline. This approach strengthens causal inference regarding the exposure-outcome relationship and is consistent with established practices in longitudinal research [53].

### Statistical analysis

The baseline characteristics of the samples were delineated using means and standard deviations for continuous variables, alongside frequencies and corresponding percentages for categorical variables. Initial comparisons between groups were conducted using chi-square tests and t-tests. A complete case analysis was used for all analyses. Participants with any missing data in the exposure variables, the outcome variable, or any of the covariates used in the multivariable models at either baseline or follow-up were excluded from the primary analysis.

To initially explore the potential shape and presence of a dose-response relationship between PA and mental health problems, we employed four-knot restricted cubic splines (RCS) to flexibly model non-linear associations. This analysis was performed for both cross-sectional (using baseline data) and longitudinal (using baseline PA and follow-up mental health outcome) relationships. For each exposure variable (MPA and VPA), the spline knots were set at the 5th, 35th, 65th, and 95th percentiles of their baseline distribution. All spline models were adjusted for the full set of covariates: age, gender, nationality, family structure, economic region, frequency of family conflict, smoking, drinking, and BMI.

We further performed logistic regression analyses to quantify the association between baseline PA and mental health problems. We conducted both cross-sectional analysis (assessing concurrent mental health status at baseline) and longitudinal analysis (assessing mental health outcomes at the 6-month follow-up). To enhance causal interpretability and control for confounding, we constructed three sequentially adjusted models: Model 1 was unadjusted; Model 2 adjusted for basic demographics (age and gender); Model 3 further included other potential confounders (nationality, family structure, economic area, family conflict, smoking, drinking, and BMI). These models quantified the association across different PA levels, reporting odds ratios (ORs) and 95% confidence intervals (CIs), using the “no PA” group as the reference. All subsequent analyses-including sensitivity tests and subgroup comparisons consistently used the fully adjusted model (Model 3) to ensure robust confounding control and result comparability.

Furthermore, we conducted a series of sensitivity analyses to verify the robustness of the results. Initially, to specifically evaluate the relationship between PA and the onset of mental health problems, we excluded participants with pre-existing mental health problems at baseline prior to initiating the analysis. Subsequently, we employed logistic regression analysis to investigate the predictive influence of baseline PA on the subsequent development of mental health problems. Additionally, to examine the potential interaction between MPA and VPA, both were incorporated into a unified analytical model (MVPA) for a comprehensive assessment. Lastly, we utilized logistic regression analysis to explore the association between baseline PA and mental health problems within the attrition group, with the objective of evaluating the potential impact of attrition bias.

Finally, we performed detailed subgroup analyses examining the relationship between MPA and VPA and mental health problems. Within these analyses, we examined multiple potential influencing factors, including age (< 13 vs. ≥ 13 years), gender (male vs. female), and economic area (affluence vs. moderate vs. poverty), to further test the robustness of our findings.

All statistical analyses were performed using R Version 4.4.1. A two-sided *P* value of less than 0.05 was considered statistically significant.

## Results

### Baseline characteristics of study participants

**Fig. 1:**
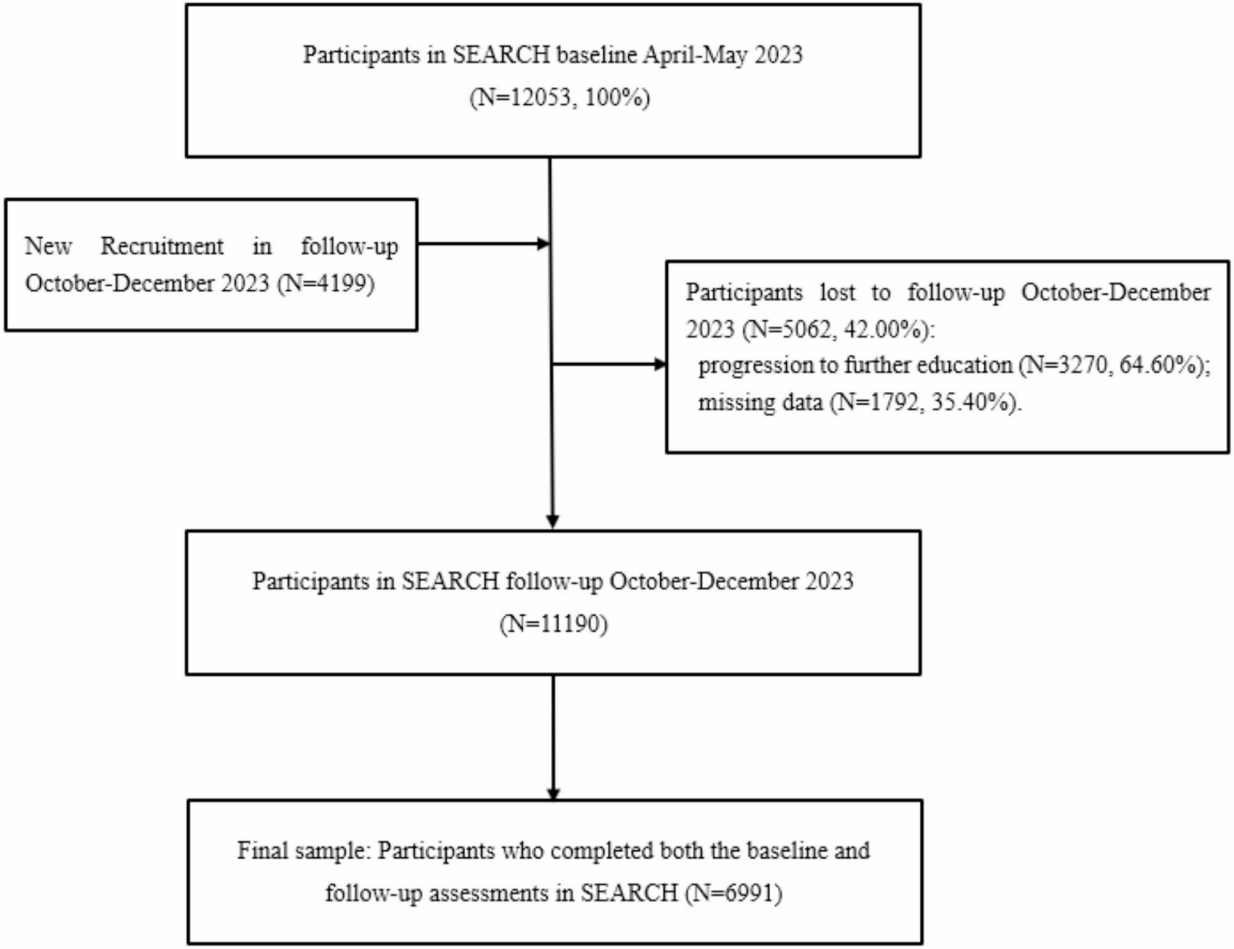
Participant flowchart of the present study

Participant flow from baseline to follow-up is detailed in Fig. 1. Of the 12,053 adolescents in the original baseline cohort, 5,062 (42.0%) were lost to follow-up. The relatively high rate of loss to follow-up mainly stems from the fact that the cohort study included adolescents in the sixth grade, the third grade of junior high school, and the third grade of senior high school. Most of them left their original surveyed schools due to normal academic advancement (such as from primary school to junior high school, from junior high school to senior high school, and from senior high school to university, etc.), making it impossible to continue the follow-up. Among the students who were lost to follow-up, 3,270 (accounting for 64.60% of the total number of lost to follow-up) were clearly unable to continue participating in the research due to reasons for further education. Consequently, the final analytical sample consisted of 6,991 adolescents (mean age = 12.71 ± 2.12 years; 53.6% male) who provide valid data at both baseline and follow-up assessments.

According to the SDQ assessment criteria, adolescents were categorized into a “healthy” group (scoring 0–15 points) and an “unhealthy” group (scoring 16–40 points). At the baseline assessment, 1,265 adolescents (18.1%) were identified as having mental health problems. Compared to the healthy group, adolescents in the unhealthy group at baseline exhibited a higher risk profile characterized by being older, lacking parental supervision, belonging to a lower socioeconomic status, experiencing more frequent family conflicts, and having a greater propensity to engage in drinking and smoking behaviors. Additionally, their BMI was also higher. Statistically significant differences were observed in the durations of MPA and VPA between the two groups (Table 1).

**Table 1:** Baseline characteristics of the study participants stratified by mental health categories (*N* = 6991)

Characteristics	Total[*N*(%)]	Categories by mental health		χ^2^/t	*P*-value
		Healthy	Unhealthy		
Gender					
Male	3748(53.6)	3096	652	2.662^a^	0.103
Female	3243(46.4)	2630	613		
Age (years)	12.71 ± 2.12	12.50 ± 2.05	13.64 ± 2.19	−16.87^b^	< 0.001
Nationality					
Han	6894(98.6)	5648	1246	0.148^a^	0.701
Non-Han	97(1.4)	78	19		
Family structure					
Both parents	5603(80.1)	4651	952	23.303^a^	< 0.001
Single parent	1069(15.3)	826	243		
Other	319(4.6)	249	70		
Economic area					
Affluence	2212(31.6)	1984	228	133.821^a^	< 0.001
Moderate	1490(21.3)	1152	338		
Poverty	3289(47.0)	2590	699		
Frequency of family conflicts					
Never	3375(48.3)	3088	287	560.837^a^	< 0.001
Sometimes	3395(48.6)	2546	849		
Always	221(3.2)	92	129		
Smoking					
Yes	397(5.7)	202	195	273.341^a^	< 0.001
No	6594(94.3)	5524	1070		
Drinking					
Yes	795(11.4)	436	359	443.271^a^	< 0.001
No	6196(88.6)	5290	906		
BMI (kg/m^2^)	19.88 ± 3.94	19.79 ± 3.99	20.26 ± 3.67	−3.40^b^	< 0.001
MPA(min/day)					
None	649(9.3)	445	204	91.836^a^	< 0.001
≤ 29	4497(64.3)	3714	783		
30–59	1339(19.2)	1145	194		
≥ 60	506(7.2)	422	84		
VPA(min/day)					
None	934(13.4)	636	298	140.152^a^	< 0.001
≤ 29	4841(69.2)	4082	759		
30–59	834(11.9)	693	141		
≥ 60	382(5.5)	315	67		

^a^: χ^2^ value

^b^: t-test value

Data were presented as frequency (%) and mean ± standard deviation

*Abbreviations*: *BMI* Body mass index, *MPA* Moderate-intensity physical activity, *VPA* Vigorous-intensity physical activity

### Implementation of restricted cubic spline models

The sample sizes between each predefined knot for the RCS models were confirmed to be sufficiently large, all significantly exceeding the recommended values based on general empirical rules. Specifically, for the cross-sectional analysis of MPA, the sample sizes at each knot were: 2221 at the first knot (5th percentile), 1512 at the second knot (35th percentile), 1413 at the third knot (65th percentile), and 1845 at the fourth knot (95th percentile). For the cross-sectional analysis of VPA, the corresponding sample sizes were 2295, 2260, 817, and 1619, respectively. The longitudinal RCS analyses for both MPA and VPA utilized the same knot locations based on the baseline distribution of PA.

### Cross-sectional analyses of different PA and mental health problems at baseline

In adjusted analyses, a negative association was identified between both MPA and VPA and baseline mental health problems, after accounting for age, gender, nationality, family structure, economic region, family conflicts, smoking, drinking and BMI.

**Fig. 2:**
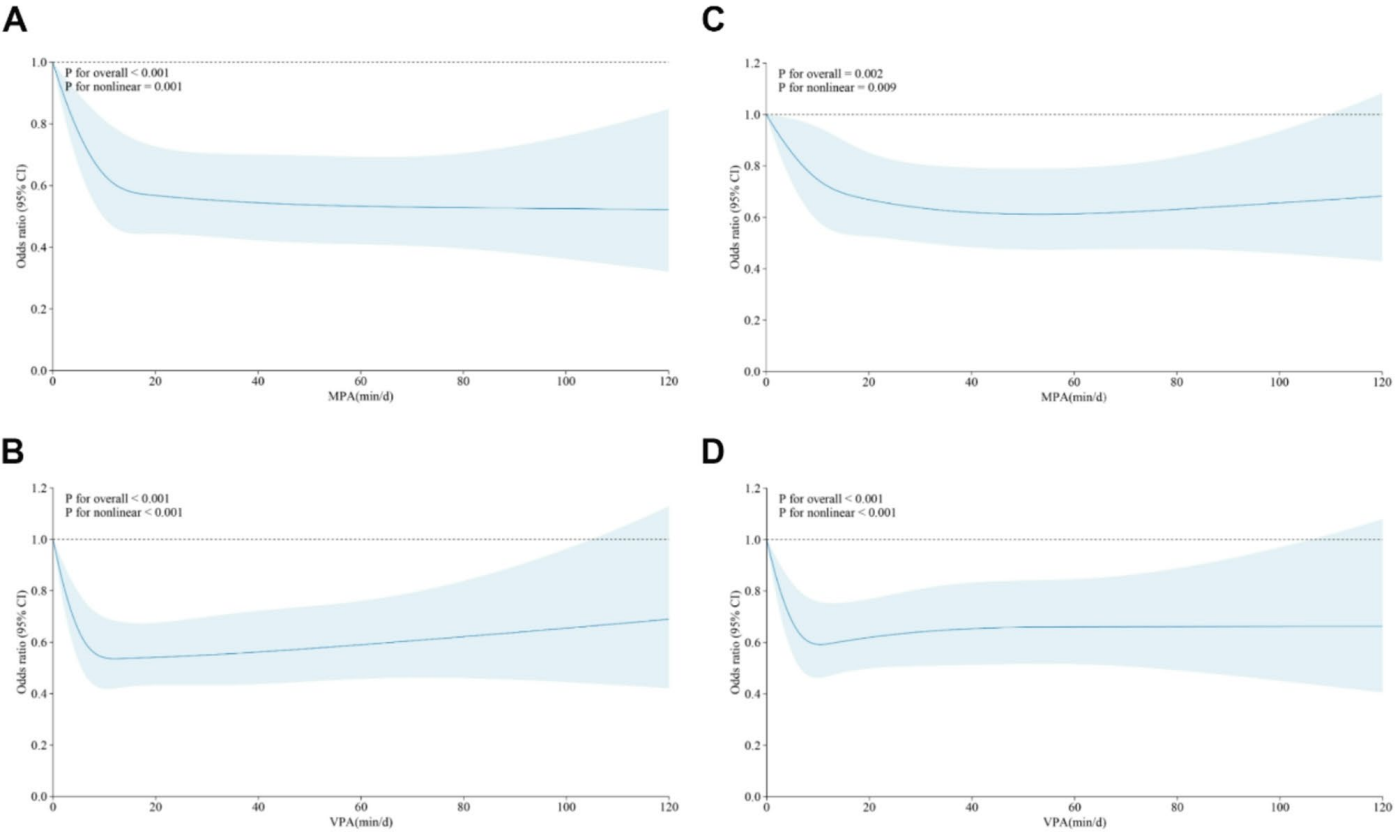
Cross-sectional and longitudinal dose-response associations between MPA and VPA and mental health problems at baseline and follow-up. Abbreviations: CI, Confidence interval; MPA, Moderate-intensity physical activity; VPA, Vigorous-intensity physical activity. Solid blue line: multivariable-adjusted odds ratios; Shading area: 95 % CIs. Multi variable models with restricted cubic splines were adjusted for age, gender, nationality, family structure, economic area, frequency of family conflicts, smoking, drinking, and BMI. A two-sided *P* value of less than 0.05 was considered statistically significant. Panel A: Dose-response relationship between MPA and mental health problems in the cross-sectional analysis at baseline, data collection time: PA (April - May 2023), Mental health (April - May 2023); Panel B: Dose-response relationship between VPA and mental health problems in the cross-sectional analysis at baseline, data collection time: PA (April - May 2023), Mental health (April - May 2023); Panel C: Dose-response relationship between MPA and subsequent mental health problems at follow-up, data collection time: PA (April - May 2023), Mental health (October - December 2023); Panel D: Dose-response relationship between VPA and subsequent mental health problems at follow-up, data collection time: PA (April - May 2023), Mental health (October - December 2023).

There was a significant negative and nonlinear relationship between the duration of MPA and baseline mental health problems (*P*-overall < 0.001, *P*-nonlinear = 0.001; Fig. 2A). The RCS curve indicated that the mental health benefits of MPA plateaued after 60 min of daily engagement, suggesting diminishing incremental benefits beyond this duration (Fig. 2A). ​Logistic regression analysis using the fully adjusted model (Model 3)​​ confirmed a consistent inverse association across all MPA duration groups (≤ 29 min/day, 30–59 min/day, ≥ 60 min/day; Table 2). Engagement in MPA for 30–59 min per day was associated with the greatest reduction in risk of mental health problems (OR = 0.436, 95% CI: 0.327–0.581; *P* < 0.001), corresponding to a 56.4% decrease in risk.

**Table 2:** Cross-sectional associations between different levels of PA and mental health problems at baseline

PA	Model 1^a^		Model 2^b^		Model 3^c^	
	OR (95% CI)	*P*-value	OR (95% CI)	*P*-value	OR (95% CI)	*P*-value
MPA(min/day)						
None	Reference		Reference		Reference	
≤ 29	0.460(0.383,0.552)	< 0.001	0.496(0.410,0.599)	< 0.001	0.566(0.445,0.718)	< 0.001
30–59	0.370(0.295,0.463)	< 0.001	0.393(0.312,0.495)	< 0.001	0.436(0.327,0.581)	< 0.001
≥ 60	0.434(0.326,0.578)	< 0.001	0.546(0.406,0.733)	< 0.001	0.573(0.398,0.824)	0.003
VPA(min/day)						
None	Reference		Reference		Reference	
≤ 29	0.397(0.339,0.465)	< 0.001	0.447(0.380,0.525)	< 0.001	0.508(0.415,0.622)	< 0.001
30–59	0.434(0.346,0.545)	< 0.001	0.496(0.393,0.626)	< 0.001	0.523(0.393,0.696)	< 0.001
≥ 60	0.454(0.337,0.611)	< 0.001	0.595(0.437,0.808)	0.001	0.657(0.458,0.944)	0.023

^a^Model 1: unadjusted for covariates

^b^Model 2: adjusted for age and gender

^c^Model 3: adjusted for age, gender, nationality, family structure, economic area, frequency of family conflicts, smoking, drinking, and BMI

*Abbreviations*: *CI* Confidence interval, *MPA* Moderate-intensity physical activity, *OR* Odds ratio, *PA* Physical activity, *VPA* Vigorous-intensity physical activity

Similarly, a significant negative nonlinear association was observed between the duration of VPA and baseline mental health problems, with overall and nonlinear *P*-values both less than 0.001 (Fig. 2B). The RCS curve indicated that the maximum benefit was achieved with up to 29 min of VPA per day. ​Logistic regression analysis based on the fully adjusted model (Model 3) confirmed a consistent protective effect across all VPA durations (Table 2). The largest risk reduction was observed for up to 29 min of VPA per day (OR = 0.508, 95% CI: 0.415–0.622; *P* < 0.001), equivalent to a 49.2% decrease in risk.

### Longitudinal associations of baseline PA with subsequent mental health problems in follow-up

Longitudinal RCS analyses revealed a significant inverse association between baseline MPA duration and the risk of mental health problems during follow-up (*P*-overall = 0.002, *P*-nonlinear = 0.009; Fig. 2C). The curve demonstrated that the probability of mental health problems decreased with increasing MPA duration, though this trend slightly reversed after 30–59 min of daily engagement (Fig. 2C). ​​​​When fully adjusted for all covariates, logistic regression analysis further corroborated these findings, showing that varying durations of baseline MPA were associated with a reduced risk of mental health problems during follow-up (Table 3). The strongest protective effect was observed in adolescents engaging in 30–59 min of MPA per day (OR = 0.548, 95% CI: 0.411–0.729), reflecting a 45.2% reduction in risk.

**Table 3:** Longitudinal associations between different levels of PA at baseline and subsequent mental health problems

PA	Model 1^a^		Model 2^b^		Model 3^c^	
	OR (95% CI)	*P*-value	OR (95% CI)	*P*-value	OR (95% CI)	*P*-value
MPA(min/day)						
None	Reference		Reference		Reference	
≤ 29	0.491(0.406,0.594)	< 0.001	0.537(0.441,0.654)	< 0.001	0.612(0.480,0.780)	< 0.001
30–59	0.459(0.365,0.577)	< 0.001	0.498(0.393,0.630)	< 0.001	0.548(0.411,0.729)	< 0.001
≥ 60	0.475(0.353,0.640)	< 0.001	0.612(0.450,0.832)	0.002	0.599(0.413,0.867)	0.007
VPA(min/day)						
None	Reference		Reference		Reference	
≤ 29	0.458(0.388,0.539)	< 0.001	0.525(0.443,0.622)	< 0.001	0.583(0.474,0.716)	< 0.001
30–59	0.501(0.396,0.633)	< 0.001	0.583(0.457,0.742)	< 0.001	0.657(0.494,0.873)	0.004
≥ 60	0.496(0.364,0.677)	< 0.001	0.668(0.484,0.921)	0.014	0.614(0.420,0.900)	0.012

^a^Model 1: unadjusted for covariates

^b^Model 2: adjusted for age and gender

^c^Model 3: adjusted for age, gender, nationality, family structure, economic area, frequency of family conflicts, smoking, drinking, and BMI

*Abbreviations*: *CI* Confidence interval, *MPA* Moderate-intensity physical activity, *OR* Odds ratio, *PA* Physical activity, *VPA* Vigorous-intensity physical activity

Data collection time: PA (April - May 2023), Mental health (October - December 2023)

Baseline VPA duration was significantly inversely correlated with the risk of mental health problems during follow-up (*P*-overall < 0.001, *P*-nonlinear < 0.001; Fig. 2D). The RCS curve indicated that the protective effect of VPA plateaued at approximately 29 min per day (Fig. 2D). Logistic regression analysis using Model 3​​ confirmed that adolescents engaging in ≤ 29 min per day of VPA experienced the most significant reduction in risk of mental health (OR = 0.583, 95% CI: 0.474–0.716), with a 41.7% decrease in the likelihood of mental health problems observed (Table 3).

### Sensitivity analyses and subgroup analyses

A series of sensitivity analyses were performed using the fully adjusted model (Model 3) to assess the robustness of the primary findings. ​​First,​​ RCS were employed to evaluate potential non-linear dose-response relationships between MPA or VPA and new-onset mental health problems in follow-up. The RCS analysis revealed no significant dose-response relationship for either activity type (*P*-overall > 0.05; *P*-nonlinear > 0.05; online Supplemental Fig. S2). Logistic regression further confirmed that the associations between baseline durations of MPA and VPA and the incidence of new-onset mental health problems during follow-up remained non-significant (all *P*-values > 0.05; online Supplemental Table S1). Second,​​ the association between baseline MVPA (a combined measure of MPA and VPA) and mental health problems was examined. Fully adjusted models showed a significant inverse association between baseline MVPA duration and mental health problems in both cross-sectional (online Supplemental Table S2) and longitudinal analyses (online Supplemental Table S3). Third, to evaluate potential attrition bias, analyses were repeated among participants lost to follow-up (*N* = 5,062). Both MPA and VPA at baseline were associated with fewer mental health problems in this subgroup, with particularly evident risk reductions at 30–59 min per day for MPA and ≤ 29 min per day for VPA. These associations remained significant after full adjustment for confounders (online Supplemental Table S4).

To explore the potential effect modification in the association between PA and mental health problems, we conducted subgroup analyses restricted to three a priori variables with strong theoretical justification: age (< 13 years vs. ≥ 13 years), gender (male vs. female) and economic area (affluence vs. moderate vs. poverty). For MPA, the association with mental health problems showed no statistically significant heterogeneity across these subgroups (all *P* values for interaction > 0.05) (online Supplemental Fig. S3). This indicates that age, gender, and economic area did not significantly moderate the relationship between MPA and mental health problems in this cohort. For VPA, the subgroup analyses revealed a potential effect modification by gender (*P* for interaction = 0.032), while no significant interactions were observed for age or economic area (*P* values > 0.05) (online Supplemental Fig. S4). Although this gender-specific finding warrants further investigation, the overall protective association between VPA and mental health problems remained consistent across all subgroups. Collectively, these results suggest that while the core relationship between PA and mental health is robust, gender may influence the strength of the association specifically for VPA.

## Discussion

In this large school-based cohort study, the duration of MPA and VPA was negatively associated with the risk of mental health problems among adolescents, demonstrating a dose-response relationship. This association appeared to reach a plateau after a certain duration threshold, indicating a plateau effect. Specifically, when compared to no physical activity, engaging in 30 to 59 min of daily MPA and no more than 29 min of daily VPA was linked to improved mental health outcomes in adolescents.

### Comparison with other studies

The findings of our study align with existing research on the positive effects of PA on adolescent mental health, while also enhancing the research design and depth of findings. Previous research, such as a nationwide study of 32,829 Finnish adolescents aged 15–16, demonstrated a dose-response relationship between leisure-time MVPA and better mental health, showing that just 30 min of activity per week could lower the risk of chronic stress symptoms by 17% [54]. Another study of Brazilian adolescents aged 12–17 found that participating in physical exercise during leisure time, regardless of how often or how long, could reduce the risk of common mental disorders by 26% [55]. However, these cross-sectional studies, while informative, generally have less causal inference power compared to our study’s longitudinal design, which is more capable of uncovering dynamic associations and potential causal pathways between variables. Additionally, our study included participants from upper elementary and middle school, extending into early high school. This broad age range facilitated the examination of potential variations in the relationship between PA and mental health across different developmental stages in adolescents, thereby surpassing the insights provided by studies that concentrate exclusively on specific age groups. This extensive age coverage allows our findings to offer a more comprehensive understanding of the relationship between PA and mental health within the adolescent population.

Conversely, other studies have reported differing conclusions. For instance, research conducted by Rothon et al. did not identify a significant association between changes in PA and alterations in depressive symptoms among adolescents in grades 7 to 9 [56]. Similarly, cohort studies conducted in the Netherlands and the UK did not furnish robust evidence supporting a clear association between PA and improvements in mental health or reductions in mental health symptoms [57, 58]. Lindwall et al. conducted over a three-year follow-up period, identified no significant correlation between alterations in PA and changes in self-esteem among girls aged 14 and 15 [59]. The inconsistency in these findings may be attributed to variations in research design, measurement instruments, sample characteristics, or the specific mental health indicators assessed.

Our key finding indicates that the relationship between PA and the risk of mental health problems in adolescents tends to stabilize after a certain duration threshold, demonstrating a “plateau effect”. Specifically, engaging in 30–59 min of MPA per day and up to 29 min of VPA per day is associated with better mental health outcomes compared to no activity. This observation is consistent with previous research suggesting that surpassing 60 min of daily exercise does not yield additional mental health benefits for adolescents [60]. Moreover, our findings align with the “J-curve” theory concerning exercise and immunosuppression, as well as the U-shaped relationship between the volume of PA and the prevalence of depressive symptoms and mental health burden, indicating that excessive exercise intensity may be potentially harmful.

### Result interpretation and research implications

Our study indicates that engaging in appropriate PA exerts a significantly positive influence on adolescent mental health. One mechanism underlying this effect is the ability of PA to enhance the secretion of various neurotransmitters, which may subsequently induce plasticity changes within the dopamine system and glutamatergic neurotransmission pathways, thereby eliciting feelings of pleasure and satisfaction [61]. Furthermore, exercise has been demonstrated to support brain health by promoting neurogenesis, enhancing synaptic plasticity, stimulating angiogenesis, and facilitating the release of vascular growth factors [62]. Considering that adolescence represents a critical period of rapid brain development, PA may be particularly effective in safeguarding mental health during this stage, as compared to adulthood [63]. Concurrently, research has shown that exercise enhances blood circulation throughout the body and brain, potentially aiding in the regulation of the hypothalamic-pituitary-adrenal (HPA) axis, which suggests that PA may contribute to mitigating physiological stress responses [64].

Although the precise mechanisms by which PA affects mental health are not yet fully elucidated, and the etiology and classification of mental health disorders such as depression and anxiety remain controversial in the scientific community, some studies have found that endorphins and other monoamines may play an important role in reducing the risk of mental health problems by activating the endocrine system, indicating that PA may also influence mental health through endocrine pathways [65]. Recent research has further revealed a novel mechanism: lactate produced by exercise can significantly increase the lactylation levels of various synaptic proteins. Specifically, the lactylation of SNAP91 promotes synaptic structural development and enhances neuronal activity in the medial prefrontal cortex (mPFC), thereby endowing the body with resilience to chronic restraint stress (CRS). In mouse models, exercise-induced lactylation of SNAP91 is crucial for preventing anxiety-like behavior after exposure to CRS. This finding provides new evidence for the metabolic adaptations occurring in the brain during exercise, particularly the phenomenon of non-histone lactylation controlling psychological functions [66].

Regarding the dose-response relationship between PA and mental health benefits, previous studies have provided preliminary evidence [67, 68], but others have pointed out that this relationship is not always a simple linear one [65]. Our research results also reveal a significant negative correlation dose-response relationship between moderate-to-vigorous levels of PA and the incidence of mental health problems. Based on this, the findings of this study support public health strategies that should focus on increasing the participation rate of adolescents in PA and promoting regular moderate intensity activity. At the same time, it is also important to recognize that beyond a certain activity level, excessively increasing duration may not be a necessary optimization strategy for obtaining additional mental health benefits.

### Strengths and limitations

This prospective cohort study possesses significant strengths. First, the large sample size of nearly 7,000 participants provides a solid foundation for drawing reliable statistical inferences. Second, the analysis incorporated a wide range of relevant factors, such as age, gender, socioeconomic status, and baseline mental health status. This not only allowed for a more nuanced exploration of the relationship between PA and adolescent mental health but also effectively controlled for potential confounding factors, thereby providing compelling evidence for the association between the two. However, the study also has several limitations. First, the geographical scope is limited to Jiangsu Province, which may restrict the generalizability of the findings to broader adolescent populations. Therefore, caution should be exercised when extrapolating these findings to formulate exercise recommendations for wider groups. Second, the study relied on self-reported data for PA and mental symptoms, introducing the risk of information bias. Self-reporting is susceptible to social desirability bias; for example, participants might overestimate their activity levels or underestimate their symptoms, leading to discrepancies between measured results and actual circumstances. Third, the relatively high rate of follow-up loss poses a potential source of bias. This was primarily because we included adolescents experiencing key academic transitions (e.g., sixth grade, third year of junior high school, third year of senior high school), where dropout often occurs in conjunction with normal academic transitions. This implies that attrition might have altered the representativeness of the sample. However, the sensitivity analyses robustly demonstrated that, even in the presence of this potential attrition bias, the association between MPA and VPA and better mental health remained quite robust, indicating that our core findings were not significantly compromised by the attrition. Furthermore, residual confounding factors cannot be entirely ruled out. Although multiple factors were controlled for, unmeasured important variables (such as general perceived stress, academic pressure, or past mental health diagnoses not captured at baseline) may still interfere with the true association between PA and mental health outcomes. Finally, while the longitudinal design provides stronger support for causal inference, completely excluding reverse causality remains challenging. That is, adolescents with better mental health may be more inclined to engage in PA. This possibility is particularly prominent in cross-sectional analyses, although the longitudinal follow-up partially mitigates this concern. Despite these limitations, the study provides strong support for the positive impact of PA on adolescent mental health.

## Conclusions

There was a notable positive association between moderate PA and improved mental health outcomes in the adolescents. Specifically, both MPA and VPA show an inverse association with the risk of mental health problems. The strongest inverse associations were observed within specific PA duration ranges—namely, 30 to 59 min per day for MPA and no more than 29 min per day for VPA. Importantly, PA durations beyond these ranges were not associated with additional mental health benefits. These findings have significant implications for public health policy and educational strategies: when promoting PA among adolescents, it is crucial to focus on the careful selection of activity types and the scientific management of activity duration.

## Supplementary Information

Supplementary Material 1.

## Data Availability

All datasets used in this study are available upon request. Interested readers can contact the corresponding author for access to the data.

## Abbreviations

BDNF: Brain-derived neurotrophic factor
BMI: Body mass index
CIs: Confidence intervals
CMD: Common mental disorders
CRS: Chronic restraint stress
DAG: Directed acyclic graph
EEG: Electroencephalogram
GHQ-12: General Health Questionnaire
HPA: Hypothalamic-pituitary-adrenal
MPA: Moderate-intensity physical activity
mPFC: Medial prefrontal cortex
MSE: Muscle-strengthening exercise
MVPA: Moderate-vigorous-intensity physical activity
OR: Odds ratio
PA: Physical activity
RCS: Restricted cubic spline
RCT: Randomized controlled trial
SDQ: Strengths and Difficulties Questionnaire
SEARCH: School-based Evaluation of Response to Advancement of Children’s Health
STROBE: Strengthening the Reporting of Observational Studies in Epidemiology
VPA: Vigorous-intensity physical activity
WHO: World Health Organization

## References

[1] Weiß M, Gutzeit J, Pryss R, Romanos M, Deserno L, Hein G. Common and differential variables of anxiety and depression in adolescence: a nation-wide smartphone-based survey. Child Adolesc Psychiatry Ment Health. 2024;18(1):103. 39153994 10.1186/s13034-024-00793-1 PMC11330155

[2] Wang K, Hu Y, He Q, Xu F, Wu YJ, Yang Y, et al. Network analysis links adolescent depression with childhood, peer, and family risk environment factors. J Affect Disord. 2023;330:165–72. 36828149 10.1016/j.jad.2023.02.103

[3] Haapala EA, Leppänen MH, Kosola S, Appelqvist-Schmidlechner K, Kraav S, Jussila JJ, et al. Childhood lifestyle behaviors and mental health symptoms in adolescence. JAMA Netw Open. 2025;8(2):e2460012. 39951263 10.1001/jamanetworkopen.2024.60012 PMC11829227

[4] Silva SA, Silva SU, Ronca DB, Gonçalves VSS, Dutra ES, Carvalho KMB. Common mental disorders prevalence in adolescents: A systematic review and meta-analyses. PLoS ONE. 2020;15(4):e0232007. 32324835 10.1371/journal.pone.0232007 PMC7179924

[5] Bao Y, Sun Y, Meng S, Shi J, Lu L. 2019-nCoV epidemic: address mental health care to empower society. Lancet. 2020;395(10224):e37–8. 32043982 10.1016/S0140-6736(20)30309-3 PMC7133594

[6] Shultz JM, Baingana F, Neria Y. The 2014 Ebola outbreak and mental health: current status and recommended response. JAMA. 2015;313(6):567–8. 25532102 10.1001/jama.2014.17934

[7] Kendler KS, Karkowski LM, Prescott CA. Causal relationship between stressful life events and the onset of major depression. Am J Psychiatry. 1999;156(6):837–41. 10360120 10.1176/ajp.156.6.837

[8] Yang L, Zhao Y, Wang Y, Liu L, Zhang X, Li B, et al. The effects of psychological stress on depression. Curr Neuropharmacol. 2015;13(4):494–504. 26412069 10.2174/1570159X1304150831150507 PMC4790405

[9] Membride H. Mental health: early intervention and prevention in children and young people. Br J Nurs. 2016;25(10):552. 27231738 10.12968/bjon.2016.25.10.552

[10] Li X, Vanderloo LM, Keown-Stoneman CDG, Cost KT, Charach A, Maguire JL, et al. Screen use and mental health symptoms in Canadian children and youth during the COVID-19 pandemic. JAMA Netw Open. 2021;4(12):e2140875. 34962557 10.1001/jamanetworkopen.2021.40875 PMC8715351

[11] Zhou SJ, Zhang LG, Wang LL, Guo ZC, Wang JQ, Chen JC, et al. Prevalence and socio-demographic correlates of psychological health problems in Chinese adolescents during the outbreak of COVID-19. Eur Child Adolesc Psychiatry. 2020;29(6):749–58. 32363492 10.1007/s00787-020-01541-4 PMC7196181

[12] Lee IM, Shiroma EJ, Lobelo F, Puska P, Blair SN, Katzmarzyk PT, Lancet Physical Activity Series Working Group. Effect of physical inactivity on major non-communicable diseases worldwide: an analysis of burden of disease and life expectancy. Lancet. 2012;380(9838):219–29. 22818936 10.1016/S0140-6736(12)61031-9 PMC3645500

[13] Herring MP, O’Connor PJ, Dishman RK. The effect of exercise training on anxiety symptoms among patients: a systematic review. Arch Intern Med. 2010;170(4):321–31. 20177034 10.1001/archinternmed.2009.530

[14] WHO Guidelines Approved by the Guidelines Review Committee. Global recommendations on physical activity for health. Geneva: World Health Organization; 2010. 26180873

[15] Aguiar EJ, Morgan PJ, Collins CE, Plotnikoff RC, Callister R. Efficacy of interventions that include diet, aerobic and resistance training components for type 2 diabetes prevention: a systematic review with meta-analysis. Int J Behav Nutr Phys Act. 2014;11:2. 24423095 10.1186/1479-5868-11-2 PMC3898566

[16] Bannuru RR, Osani MC, Vaysbrot EE, Arden NK, Bennell K, Bierma-Zeinstra SMA, et al. OARSI guidelines for the non-surgical management of knee, hip, and polyarticular osteoarthritis. Osteoarthritis Cartilage. 2019;27(11):1578–89. 31278997 10.1016/j.joca.2019.06.011

[17] Alghadir AH, Aly FA, Gabr SA. Effect of moderate aerobic training on bone metabolism indices among adult humans. Pak J Med Sci. 2014;30(4):840–4. 25097528 10.12669/pjms.304.4624 PMC4121709

[18] Landi F, Marzetti E, Martone AM, Bernabei R, Onder G. Exercise as a remedy for sarcopenia. Curr Opin Clin Nutr Metab Care. 2014;17(1):25–31. 24310054 10.1097/MCO.0000000000000018

[19] McVey Neufeld SF, Ahn M, Kunze WA, McVey Neufeld KA. Adolescence, the Microbiota-Gut-Brain Axis, and the emergence of psychiatric disorders. Biol Psychiatry. 2024;95(4):310–8. 37839790 10.1016/j.biopsych.2023.10.006

[20] Beauchamp MR, Puterman E, Lubans DR. Physical inactivity and mental health in late adolescence. JAMA Psychiatry. 2018;75(6):543–4. 29710114 10.1001/jamapsychiatry.2018.0385

[21] De Moor MH, Beem AL, Stubbe JH, Boomsma DI, De Geus EJC. Regular exercise, anxiety, depression and personality: a population-based study. Prev Med. 2006;42(4):273–9. 16439008 10.1016/j.ypmed.2005.12.002

[22] Kantomaa MT, Tammelin TH, Demakakos P, Ebeling HE, Taanila AM. Physical activity, emotional and behavioural problems, maternal education and self-reported educational performance of adolescents. Health Educ Res. 2010;25(2):368–79. 19762353 10.1093/her/cyp048

[23] Valois RF, Zullig KJ, Huebner ESt, Drane JW. Physical activity behaviors and perceived life satisfaction among public high school adolescents. J Sch Health. 2004;74(2):59–65. 15077500 10.1111/j.1746-1561.2004.tb04201.x

[24] Burns RD, Kim Y, Fu Y, Byun W, Bai Y. Independent and joint associations of aerobic and muscle-strengthening exercise with mental health in adolescents: A cross-sectional analysis before and during COVID-19 using the 2015–2021 National youth risk behavior survey. Prev Med. 2023;177:107750. 37918448 10.1016/j.ypmed.2023.107750

[25] Philippot A, Dubois V, Lambrechts K, Grogna D, Robert A, Jonckheer U, et al. Data on the impact of physical exercise treatment on depression and anxiety in a psychiatric hospital for adolescents. Data Brief. 2022;42:108165. 35496473 10.1016/j.dib.2022.108165 PMC9046950

[26] Xu W, Shen W, Wang S. Intervention of adolescent’ mental health during the outbreak of COVID-19 using aerobic exercise combined with acceptance and commitment therapy. Child Youth Serv Rev. 2021;124:105960. 36567871 10.1016/j.childyouth.2021.105960 PMC9757822

[27] Gu Q, Zhao X, Lin L, Teo WP, Liu L, Yuan S. Effects of open-skill and closed-skill exercise on subthreshold depression in female adolescents: A randomized controlled trial. Int J Clin Health Psychol. 2024;24(4):100512. 39659958 10.1016/j.ijchp.2024.100512 PMC11630631

[28] Kandola A, Cruz BDP, Hayes JF, Owen N, Dunstan DW, Hallgren M. Impact on adolescent mental health of replacing screen-use with exercise: A prospective cohort study. J Affect Disord. 2022;301:240–7. 34999126 10.1016/j.jad.2021.12.064

[29] Wu J, Shao Y, Zang W, Hu J. Is physical exercise associated with reduced adolescent social anxiety mediated by psychological resilience? Evidence from a longitudinal multi-wave study in China. Child Adolesc Psychiatry Ment Health. 2025;19(1):17. 40045423 10.1186/s13034-025-00867-8 PMC11884043

[30] Perron RM, Graham CA, Feldman JR, Moffett RA, Hall EE. Do exergames allow children to achieve physical activity intensity commensurate with National guidelines? Int J Exerc Sci. 2011;4(4):257–64. 27182367 10.70252/UHDV1774 PMC4738919

[31] DiGiacomo JC, Angus LDG, Cardozo-Stolberg S, Wallace R, Gerber N, Munnangi S, et al. Betwixt and between: a surgical post-acute treatment unit (SPA) for the optimal care of elderly patients with isolated hip fractures. Aging Clin Exp Res. 2019;31(12):1743–53. 30968288 10.1007/s40520-019-01119-4

[32] Jäger A, Pieper A, Priebe K, Hellweg R, Meyer K, Herrmann S, et al. Effects of high intensity interval training on serum brain-derived neurotrophic factor in individuals with PTSD. J Psychiatr Res. 2024;180:355–61. 39520767 10.1016/j.jpsychires.2024.11.009

[33] Wang X, Cai ZD, Jiang WT, Fang YY, Sun WX, Wang X. Systematic review and meta-analysis of the effects of exercise on depression in adolescents. Child Adolesc Psychiatry Ment Health. 2022;16(1):16. 35227300 10.1186/s13034-022-00453-2 PMC8886903

[34] Cooney GM, Dwan K, Greig CA, Lawlor DA, Rimer J, Waugh FR, et al. Exercise for depression. Cochrane Database Syst Rev. 2013;2013(9):CD004366. 24026850 10.1002/14651858.CD004366.pub6 PMC9721454

[35] Kvam S, Kleppe CL, Nordhus IH, Hovland A. Exercise as a treatment for depression: A meta-analysis. J Affect Disord. 2016;202:67–86. 27253219 10.1016/j.jad.2016.03.063

[36] Schuch FB, Vancampfort D, Richards J, Rosenbaum S, Ward PB, Stubbs B. Exercise as a treatment for depression: A meta-analysis adjusting for publication bias. J Psychiatr Res. 2016;77:42–51. 26978184 10.1016/j.jpsychires.2016.02.023

[37] Chalder M, Wiles NJ, Campbell J, Hollinghurst SP, Haase AM, Taylor AH, et al. Facilitated physical activity as a treatment for depressed adults: randomised controlled trial. BMJ. 2012;344:e2758. 22674921 10.1136/bmj.e2758 PMC3368484

[38] Toseeb U, Brage S, Corder K, Dunn VJ, Jones PB, Owens M, et al. Exercise and depressive symptoms in adolescents: a longitudinal cohort study. JAMA Pediatr. 2014;168(12):1093–100. 25317674 10.1001/jamapediatrics.2014.1794

[39] Mbithi G, Mabrouk A, Sarki A, Odhiambo R, Namuguzi M, Dzombo JT, et al. Mental health and psychological well-being of Kenyan adolescents from Nairobi and the Coast regions in the context of COVID-19. Child Adolesc Psychiatry Ment Health. 2023;17(1):63. 37208781 10.1186/s13034-023-00613-y PMC10198601

[40] Piechaczek CE, Greimel E, Feldmann L, Pehl V, Allgaier AK, Frey M, et al. Interactions between FKBP5 variation and environmental stressors in adolescent major depression. Psychoneuroendocrinology. 2019;106:28–37. 30953930 10.1016/j.psyneuen.2019.03.025

[41] Buchan MC, Romano I, Butler A, Laxer RE, Patte KA, Leatherdale ST. Bi-directional relationships between physical activity and mental health among a large sample of Canadian youth: a sex-stratified analysis of students in the COMPASS study. Int J Behav Nutr Phys Act. 2021;18(1):132. 34627283 10.1186/s12966-021-01201-z PMC8501578

[42] Kandola A, Lewis G, Osborn DPJ, Stubbs B, Hayes JF. Depressive symptoms and objectively measured physical activity and sedentary behaviour throughout adolescence: a prospective cohort study. Lancet Psychiatry. 2020;7(3):262–71. 32059797 10.1016/S2215-0366(20)30034-1 PMC7033559

[43] Viner R, Russell S, Saulle R, Croker H, Stansfield C, Packer J, et al. School closures during social lockdown and mental health, health Behaviors, and Well-being among children and adolescents during the first COVID-19 wave: A systematic review. JAMA Pediatr. 2022;176(4):400–9. 35040870 10.1001/jamapediatrics.2021.5840

[44] Hofman A, Voortman T, Ikram MA, Luik AI. Substitutions of physical activity, sedentary behaviour and sleep: associations with mental health in middle-aged and elderly persons. J Epidemiol Community Health. 2022;76(2):175–81. 34301796 10.1136/jech-2020-215883 PMC8762024

[45] Oftedal S, Kolt GS, Holliday EG, Stamatakis E, Vandelanotte C, Brown WJ, et al. Associations of health-behavior patterns, mental health and self-rated health. Prev Med. 2019;118:295–303. 30476503 10.1016/j.ypmed.2018.11.017

[46] van Sluijs EMF, Ekelund U, Crochemore-Silva I, Guthold R, Ha A, Lubans D, et al. Physical activity behaviours in adolescence: current evidence and opportunities for intervention. Lancet. 2021;398(10298):429–42. 34302767 10.1016/S0140-6736(21)01259-9 PMC7612669

[47] Zhang R, Wang Y, Womer F, Yang W, Wang X, Xu X, et al. School-based evaluation advancing response for child health (SEARCH): a mixed longitudinal cohort study from multifaceted perspectives in Jiangsu, China. BMJ Ment Health. 2023;26(1):e300861. 37907330 10.1136/bmjment-2023-300861 PMC10618980

[48] Zhao J, Yu Z, Sun X, Wu S, Zhang J, Zhang D, et al. Association between screen time trajectory and early childhood development in children in China. JAMA Pediatr. 2022;176(8):768–75. 35666518 10.1001/jamapediatrics.2022.1630 PMC9171655

[49] Goodman R. Psychometric properties of the strengths and difficulties questionnaire. J Am Acad Child Adolesc Psychiatry. 2001;40(11):1337–45. 11699809 10.1097/00004583-200111000-00015

[50] Kinyanda E, Kizza R, Abbo C, Ndyanabangi S, Levin J. Prevalence and risk factors of depression in childhood and adolescence as seen in four districts of North-Eastern Uganda. BMC Int Health Hum Rights. 2013;13:19. 23561039 10.1186/1472-698X-13-19 PMC3626891

[51] Bannink R, Broeren S, Zwanenburg EJ, van As E, van de Looij-Jansen P, Raat H. Effectiveness of a Web-based tailored intervention (E-health4Uth) and consultation to promote adolescents’ health: randomized controlled trial. J Med Internet Res. 2014;16(5):e143. 24878521 10.2196/jmir.3163 PMC4060146

[52] Chen Q, Tao J, Liu Z, JIN Q. Association between screen time and sleep States among preschool children of different genders in Zunyi area of Guizhou. J Guizhou Med Univ. 2025;50(2):275–80.

[53] Loh WW, Ren D. Adjusting for baseline measurements of the mediators and outcome as a first step toward eliminating confounding biases in mediation analysis. Perspect Psychol Sci. 2023;18(5):1254–66. 36749872 10.1177/17456916221134573

[54] Jussila JJ, Pulakka A, Ervasti J, Halonen JI, Mikkonen S, Allaouat S, et al. Associations of leisure-time physical activity and active school transport with mental health outcomes: A population-based study. Scand J Med Sci Sports. 2023;33(5):670–81. 36571113 10.1111/sms.14292

[55] Ferreira VR, Jardim TV, Póvoa TIR, Viana RB, Sousa ALL, Jardim PV. Physical inactivity during leisure and school time is associated with the presence of common mental disorders in adolescence. Rev Saude Publica. 2020;54:128. 33295594 10.11606/s1518-8787.2020054001888 PMC7688259

[56] Rothon C, Edwards P, Bhui K, Viner RM, Taylor S, Stansfeld SA. Physical activity and depressive symptoms in adolescents: a prospective study. BMC Med. 2010;8:32. 20509868 10.1186/1741-7015-8-32 PMC2895574

[57] Van Dijk ML, Savelberg HHCM, Verboon P, Kirschner PA, De Groot RHM. Decline in physical activity during adolescence is not associated with changes in mental health. BMC Public Health. 2016;16:300. 27056368 10.1186/s12889-016-2983-3 PMC4825085

[58] Bell SL, Audrey S, Gunnell D, Cooper A, Campbell R. The relationship between physical activity, mental wellbeing and symptoms of mental health disorder in adolescents: a cohort study. Int J Behav Nutr Phys Act. 2019;16(1):138. 31878935 10.1186/s12966-019-0901-7 PMC6933715

[59] Lindwall M, Asci H, Crocker P. The physical self in motion: within-person change and associations of change in self-esteem, physical self-concept, and physical activity in adolescent girls. J Sport Exerc Psychol. 2014;36(6):551–63. 25602138 10.1123/jsep.2013-0258

[60] Harvey SB, Øverland S, Hatch SL, Wessely S, Mykletun A, Hotopf M. Exercise and the prevention of depression: results of the HUNT cohort study. Am J Psychiatry. 2018;175(1):28–36. 28969440 10.1176/appi.ajp.2017.16111223

[61] Kuwamizu R, Yamada Y. Physiological mechanism of acute exercise benefits for human cognition: possible involvement of dopamine release and central command. J Physiol. 2024;602(6):997–9. 38412050 10.1113/JP286234

[62] van Praag H. Exercise and the brain: something to Chew on. Trends Neurosci. 2009;32(5):283–90. 19349082 10.1016/j.tins.2008.12.007 PMC2680508

[63] Hillman CH, Buck SM, Themanson JR, Pontifex MB, Castelli DM. Aerobic fitness and cognitive development: Event-related brain potential and task performance indices of executive control in preadolescent children. Dev Psychol. 2009;45(1):114–29. 19209995 10.1037/a0014437

[64] Guszkowska M. Effects of exercise on anxiety, depression and mood. Psychiatr Pol. 2004;38(4):611–20. 15518309

[65] Liu J, Ji M, Clarke CV, Liu R, Ma X, An R. Physical activity and mental health among Chinese adolescents. Am J Health Behav. 2021;45(2):309–22. 33888191 10.5993/AJHB.45.2.10

[66] Yan L, Wang Y, Hu H, Yang D, Wang W, Luo Z, et al. Physical exercise mediates cortical synaptic protein lactylation to improve stress resilience. Cell Metab. 2024;36(9):2104–e21174. 39163863 10.1016/j.cmet.2024.07.018

[67] Hamer M, Stamatakis E, Steptoe A. Dose-response relationship between physical activity and mental health: the Scottish health survey. Br J Sports Med. 2009;43(14):1111–4. 18403415 10.1136/bjsm.2008.046243

[68] Khan Y, Taghdisi MH, Nourijelyani K. Psychological Well-Being (PWB) of school adolescents aged 12–18 year, its correlation with general levels of physical activity (PA) and Socio-Demographic factors in Gilgit, Pakistan. Iran J Public Health. 2015;44(6):804–13. 26258093 PMC4524305

