# Supplementary material for: Non-linear dose-response association between physical activity and mental health in adolescents: a prospective cohort study based on SEARCH
Source: Int J Behav Nutr Phys Act. 2025 Nov 20;22:146. doi: 10.1186/s12966-025-01848-y (PMC12632063; doi:10.1186/s12966-025-01848-y)
Supplement: Supplementary file 1 — Supplementary Material 1. [file 12966_2025_1848_MOESM1_ESM.docx]

**Supplemental Materials**

**Checklist**. Strengthening the Reporting of Observational Studies in Epidemiology (STROBE)

**Figure S1**. Selection of adjustment covariates by directed acyclic graph

**Figure S2**. Longitudinal dose-response associations between MPA and VPA and new-onset mental health problems

**Figure S3**. Subgroup analyses of the association between MPA and the risk of mental health problems

**Figure S4**. Subgroup analyses of the association between VPA and the risk of mental health problems

**Table S1**. Longitudinal associations between different PA at baseline and new-onset mental health problems during the follow-up period

**Table S2**. Cross-sectional associations between MVPA and mental health problems at baseline

**Table S3**. Longitudinal associations between MVPA at baseline and subsequent mental health problems during the follow-up period

**Table S4**. Cross-sectional associations between different levels of PA and baseline mental health problems in the adolescents lost to follow-up (N=5062).

**Checklist**. Strengthening the Reporting of Observational Studies in Epidemiology (STROBE)

|  | Item No. | Recommendation | Page  No. | Relevant text from manuscript |
| --- | --- | --- | --- | --- |
| **Title and abstract** | 1 | (*a*) Indicate the study’s design with a commonly used term in the title or the abstract | 1,2 |  |
|  |  | (*b*) Provide in the abstract an informative and balanced summary of what was done and what was found | 1,2 |  |
| Introduction | | | |  |
| Background/rationale | 2 | Explain the scientific background and rationale for the investigation being reported | 2-5 |  |
| Objectives | 3 | State specific objectives, including any prespecified hypotheses | 4-5 |  |
| Methods | | | |  |
| Study design | 4 | Present key elements of study design early in the paper | 5 |  |
| Setting | 5 | Describe the setting, locations, and relevant dates, including periods of recruitment, exposure, follow-up, and data collection | 5 |  |
| Participants | 6 | (*a*) *Cohort study*—Give the eligibility criteria, and the sources and methods of selection of participants. Describe methods of follow-up  *Case-control study*—Give the eligibility criteria, and the sources and methods of case ascertainment and control selection. Give the rationale for the choice of cases and controls  *Cross-sectional study*—Give the eligibility criteria, and the sources and methods of selection of participants | 5 |  |
| Variables | 7 | Clearly define all outcomes, exposures, predictors, potential confounders, and effect modifiers. Give diagnostic criteria, if applicable | 6,7 |  |
| Data sources/ measurement | 8* | For each variable of interest, give sources of data and details of methods of assessment (measurement). Describe comparability of assessment methods if there is more than one group | 6,7 |  |
| Bias | 9 | Describe any efforts to address potential sources of bias | 7,8 |  |
| Study size | 10 | Explain how the study size was arrived at | 5 |  |

Continued on next page

| Quantitative variables | 11 | Explain how quantitative variables were handled in the analyses. If applicable, describe which groupings were chosen and why | 6,7 |  |
| --- | --- | --- | --- | --- |
| Statistical methods | 12 | (*a*) Describe all statistical methods, including those used to control for confounding | 7,8 |  |
|  |  | (*b*) Describe any methods used to examine subgroups and interactions | 7,8 |  |
|  |  | (*c*) Explain how missing data were addressed | 7,8 |  |
|  |  | (*d*) *Cohort study*—If applicable, explain how loss to follow-up was addressed  *Case-control study*—If applicable, explain how matching of cases and controls was addressed  *Cross-sectional study*—If applicable, describe analytical methods taking account of sampling strategy | 5 |  |
|  |  | (*e*) Describe any sensitivity analyses | 7,8 |  |
| Results | | | | |
| Participants | 13* | (a) Report numbers of individuals at each stage of study—eg numbers potentially eligible, examined for eligibility, confirmed eligible, included in the study, completing follow-up, and analysed | 8 |  |
|  |  | (b) Give reasons for non-participation at each stage | 8 |  |
|  |  | (c) Consider use of a flow diagram | 26, Fig.1 |  |
| Descriptive data | 14* | (a) Give characteristics of study participants (eg demographic, clinical, social) and information on exposures and potential confounders | 8 |  |
|  |  | (b) Indicate number of participants with missing data for each variable of interest |  |  |
|  |  | (c) *Cohort study*—Summarise follow-up time (eg, average and total amount) |  |  |
| Outcome data | 15* | *Cohort study*—Report numbers of outcome events or summary measures over time | 8 |  |
|  |  | *Case-control study—*Report numbers in each exposure category, or summary measures of exposure |  |  |
|  |  | *Cross-sectional study—*Report numbers of outcome events or summary measures |  |  |
| Main results | 16 | (*a*) Give unadjusted estimates and, if applicable, confounder-adjusted estimates and their precision (eg, 95% confidence interval). Make clear which confounders were adjusted for and why they were included | 9,10 |  |
|  |  | (*b*) Report category boundaries when continuous variables were categorized |  |  |
|  |  | (*c*) If relevant, consider translating estimates of relative risk into absolute risk for a meaningful time period |  |  |

Continued on next page

| Other analyses | 17 | Report other analyses done—eg analyses of subgroups and interactions, and sensitivity analyses | 10 |  |
| --- | --- | --- | --- | --- |
| Discussion | | | | |
| Key results | 18 | Summarise key results with reference to study objectives | 10,11 |  |
| Limitations | 19 | Discuss limitations of the study, taking into account sources of potential bias or imprecision. Discuss both direction and magnitude of any potential bias | 12,13 |  |
| Interpretation | 20 | Give a cautious overall interpretation of results considering objectives, limitations, multiplicity of analyses, results from similar studies, and other relevant evidence | 13 |  |
| Generalisability | 21 | Discuss the generalisability (external validity) of the study results | 13 |  |
| Other information | |  | | |
| Funding | 22 | Give the source of funding and the role of the funders for the present study and, if applicable, for the original study on which the present article is based | 15 |  |

*Give information separately for cases and controls in case-control studies and, if applicable, for exposed and unexposed groups in cohort and cross-sectional studies.

**Note:** An Explanation and Elaboration article discusses each checklist item and gives methodological background and published examples of transparent reporting. The STROBE checklist is best used in conjunction with this article (freely available on the Web sites of PLoS Medicine at http://www.plosmedicine.org/, Annals of Internal Medicine at http://www.annals.org/, and Epidemiology at http://www.epidem.com/). Information on the STROBE Initiative is available at www.strobe-statement.org.

**Figure S1**. Selection of adjustment covariates by directed acyclic graph


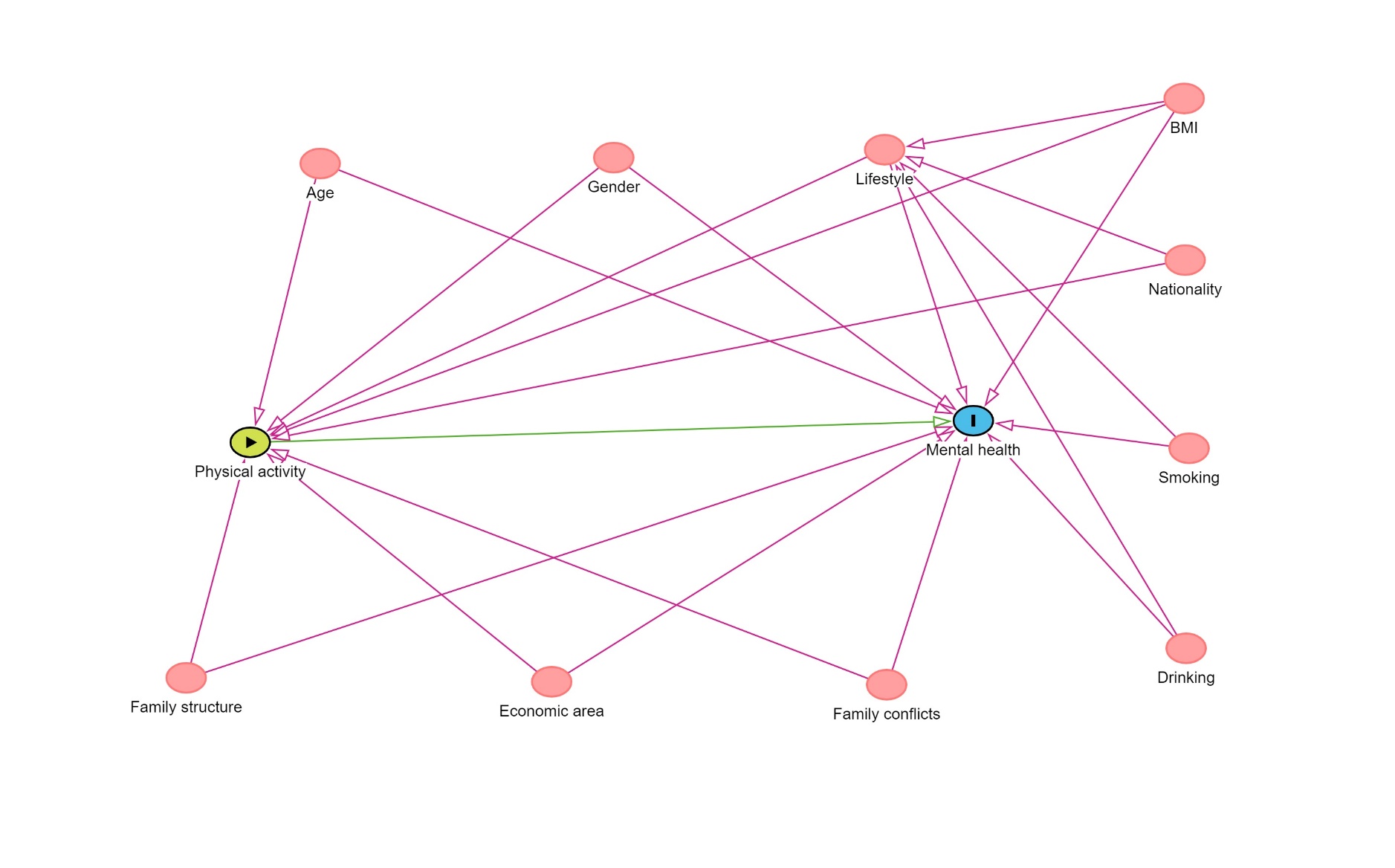


Green circle with a triangle inside: exposure; blue circle with a bar inside: outcome; pink circle: potential confounder that has been adjusted; pink line: biasing path; green line: causal path.

**Abbreviations**: BMI, body mass index

**Figure S2.** Longitudinal dose-response associations between MPA and VPA and new-onset mental health problems


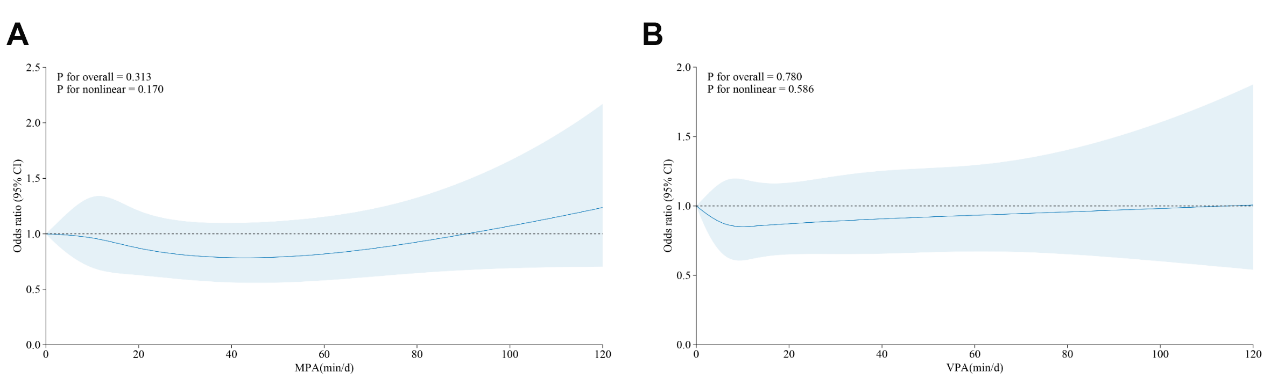


**Abbreviations:** CI, confidence interval; MPA, moderate-intensity physical activity; VPA, vigorous-intensity physical activity.

Solid blue line: multivariable-adjusted odds ratios; Shading area: 95 % CIs. Multi variable models with restricted cubic splines were adjusted for age, gender, nationality, family structure, economic area, frequency of family conflicts, smoking, drinking, and BMI. A two-sided *P* value of less than 0.05 was considered statistically significant. Panel A: The longitudinal dose-response associations between MPA and new-onset mental health problems in adolescents; Panel B: The longitudinal dose-response associations between VPA and new-onset mental health problems in adolescents.

**Figure S3**. Subgroup analyses of the association between MPA and the risk of mental health problems

**
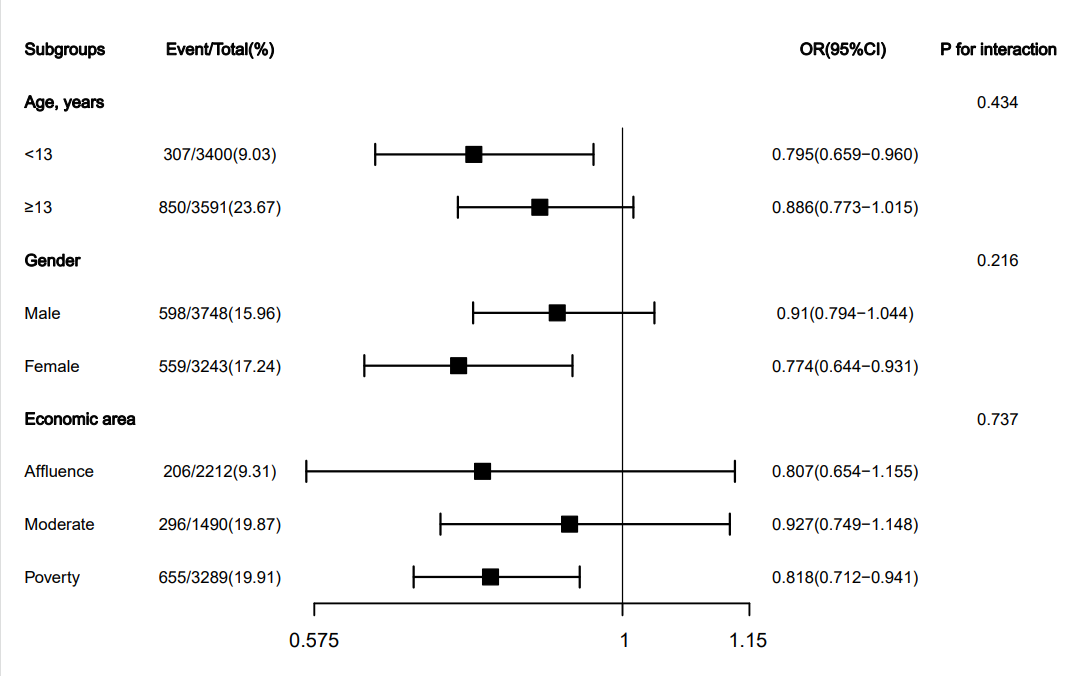
**

**Abbreviations**: CI, confidence interval; MPA, moderate-intensity physical activity; OR, odd ratio

Association between MPA and subsequent mental health stratified by different factors. Graphs show ORs and 95% CIs for subsequent mental health adjusted for age, gender and economic area. The strata variable was not included in the model when stratifying by itself.


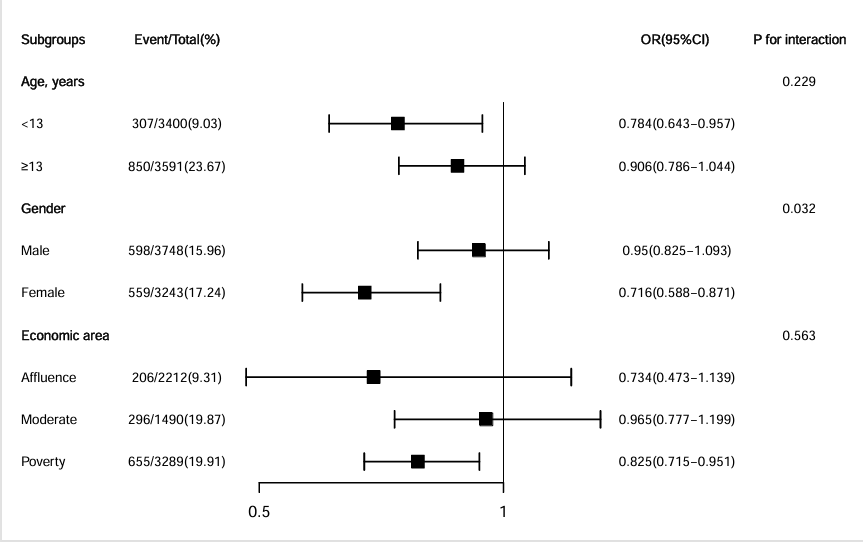
**Figure S4**. Subgroup analyses of the association between VPA and the risk of mental health problems

**Abbreviations**: CI, confidence interval; VPA, vigorous-intensity physical activity; OR, odd ratio

Association between VPA and subsequent mental health stratified by different factors. Graphs show ORs and 95% CIs for subsequent mental health adjusted for age, gender and economic area. The strata variable was not included in the model when stratifying by itself.

**Table S1.** Longitudinal associations between different PA at baseline and new-onset mental health problems during the follow-up period

| **PA** | **Model 1^a^** | |  | **Model 2^b^** | |  | **Model 3^c^** | |
| --- | --- | --- | --- | --- | --- | --- | --- | --- |
|  | **OR (95% CI)** | ***P*-value** |  | **OR (95% CI)** | ***P*-value** |  | **OR (95% CI)** | ***P*-value** |
| **MPA**(min/day) |  |  |  |  |  |  |  |  |
| None | Reference |  |  | Reference |  |  | Reference |  |
| ≤29 | 0.647(0.484,0.865) | 0.003 |  | 0.710(0.530,0.953) | 0.022 |  | 0.779(0.551,1.101) | 0.157 |
| 30-59 | 0.678(0.482,0.954) | 0.026 |  | 0.740(0.524,1.043) | 0.086 |  | 0.798(0.536,1.189) | 0.268 |
| ≥60 | 0.716(0.465,1.104) | 0.131 |  | 0.877(0.565,1.361) | 0.558 |  | 0.869(0.525,1.438) | 0.585 |
| **VPA**(min/day) |  |  |  |  |  |  |  |  |
| None | Reference |  |  | Reference |  |  | Reference |  |
| ≤29 | 0.699(0.542,0.901) | 0.006 |  | 0.792(0.612,1.025) | 0.076 |  | 0.841(0.624,1.134) | 0.256 |
| 30-59 | 0.705(0.493,1.009) | 0.056 |  | 0.803(0.559,1.154) | 0.235 |  | 0.827(0.549,1.245) | 0.363 |
| ≥60 | 0.790(0.503,1.242) | 0.308 |  | 0.993(0.626,1.574) | 0.975 |  | 0.948(0.564,1.592) | 0.839 |

^a^Model 1: unadjusted for covariates.

^b^Model 2: adjusted for age and gender.

^c^Model 3: adjusted for age, gender, nationality, family structure, economic area, frequency of family conflicts, smoking, drinking, and BMI.

**Abbreviations:** CI, confidence interval; MPA, moderate-intensity physical activity; OR, odds ratio; PA, physical activity; VPA, vigorous-intensity physical activity

**Table S2.** Cross-sectional associations between MVPA and mental health problems at baseline

|  | **Model 1^a^** | |  | **Model 2^b^** | |  | **Model 3^c^** | |
| --- | --- | --- | --- | --- | --- | --- | --- | --- |
|  | **OR (95% CI)** | ***P*-value** |  | **OR (95% CI)** | ***P*-value** |  | **OR (95% CI)** | ***P*-value** |
| **MVPA**(min/day) | |  |  |  |  |  |  |  |
| None | Reference |  |  | Reference |  |  | Reference |  |
| ≤29 | 0.454(0.367,0.562) | <0.001 |  | 0.515(0.413,0.642) | <0.001 |  | 0.616(0.465,0.817) | 0.001 |
| 30-59 | 0.308(0.244,0.388) | <0.001 |  | 0.357 (0.281,0.454) | <0.001 |  | 0.428(0.316,0.579) | <0.001 |
| ≥60 | 0.356(0.279,0.453) | <0.001 |  | 0.438(0.341,0.563) | <0.001 |  | 0.536(0.392,0.733) | <0.001 |

^a^Model 1: unadjusted for covariates.

^b^Model 2: adjusted for age and gender.

^c^Model 3: adjusted for age, gender, nationality, family structure, economic area, frequency of family conflicts, smoking, drinking, and BMI.

**Abbreviations:** CI, confidence interval; MVPA, moderate-vigorous-intensity physical activity; OR, odds ratio; PA, physical activity

**Table S3.** Longitudinal associations between MVPA at baseline and subsequent mental health problems during the follow-up period

|  | **Model 1^a^** | |  | **Model 2^b^** | |  | **Model 3^c^** | |
| --- | --- | --- | --- | --- | --- | --- | --- | --- |
|  | **OR (95% CI)** | ***P*-value** |  | **OR (95% CI)** | ***P*-value** |  | **OR (95% CI)** | ***P*-value** |
| **MVPA**(min/day) | |  |  |  |  |  |  |  |
| None | Reference |  |  | Reference |  |  | Reference |  |
| ≤29 | 0.497 (0.398,0.620) | <0.001 |  | 0.576(0.457,0.725) | <0.001 |  | 0.614(0.462,0.816) | 0.001 |
| 30-59 | 0.364(0.286,0.463) | <0.001 |  | 0.433 (0.338,0.556) | <0.001 |  | 0.480(0.354,0.649) | <0.001 |
| ≥60 | 0.423(0.329,0.543) | <0.001 |  | 0.537(0.415,0.696) | <0.001 |  | 0.576(0.420,0.788) | 0.001 |

^a^Model 1: unadjusted for covariates.

^b^Model 2: adjusted for age and gender.

^c^Model 3: adjusted for age, gender, nationality, family structure, economic area, frequency of family conflicts, smoking, drinking, and BMI.

**Abbreviations:** CI, confidence interval; MVPA, moderate-vigorous-intensity physical activity; OR, odds ratio; PA, physical activity

**Table S4.** Cross-sectional associations between different levels of PA and baseline mental health problems in the adolescents lost to follow-up (N=5062)

| **PA** | **Model 1^a^** | |  | **Model 2^b^** | |  | **Model 3^c^** | |
| --- | --- | --- | --- | --- | --- | --- | --- | --- |
|  | **OR (95% CI)** | ***P*-value** |  | **OR (95% CI)** | ***P*-value** |  | **OR (95% CI)** | ***P*-value** |
| **MPA**(min/day) |  |  |  |  |  |  |  |  |
| None | Reference |  |  | Reference |  |  | Reference |  |
| ≤29 | 0.462(0.386,0.554) | <0.001 |  | 0.461(0.384,0.553) | <0.001 |  | 0.489(0.392,0.609) | <0.001 |
| 30-59 | 0.351(0.275,0.447) | <0.001 |  | 0.383(0.299,0.489) | <0.001 |  | 0.391(0.294,0.520) | <0.001 |
| ≥60 | 0.473(0.349,0.640) | <0.001 |  | 0.534(0.393,0.726) | <0.001 |  | 0.505(0.352,0.724) | <0.001 |
| **VPA**(min/day) |  |  |  |  |  |  |  |  |
| None | Reference |  |  | Reference |  |  | Reference |  |
| ≤29 | 0.445(0.384,0.516) | <0.001 |  | 0.479(0.413,0.557) | <0.001 |  | 0.508(0.427,0.605) | <0.001 |
| 30-59 | 0.464(0.416,0.518) | <0.001 |  | 0.499(0.385,0.648) | <0.001 |  | 0.532(0.412,0.586) | <0.001 |
| ≥60 | 0.486(0.354,0.667) | <0.001 |  | 0.600(0.434,0.831) | <0.001 |  | 0.561(0.385,0.818) | 0.003 |

^a^Model 1: unadjusted for covariates.

^b^Model 2: adjusted for age and gender.

^c^Model 3: adjusted for age, gender, nationality, family structure, economic area, frequency of family conflicts, smoking, drinking, and BMI.

**Abbreviations:** CI, Confidence interval; MPA, moderate-intensity physical activity; OR, odds ratio; PA, physical activity; VPA, vigorous-intensity physical activity
